# Diversity in connexin biology

**DOI:** 10.1016/j.jbc.2023.105263

**Published:** 2023-09-20

**Authors:** Sergiu A. Lucaciu, Stephanie E. Leighton, Alexandra Hauser, Ryan Yee, Dale W. Laird

**Affiliations:** 1 Department of Anatomy and Cell Biology, University of Western Ontario, London, Ontario, Canada; 2 Department of Physiology and Pharmacology, University of Western Ontario, London, Ontario, Canada

**Keywords:** connexin, gap junctions, gap junctional intercellular communication, expression, human, noncanonical, canonical, hemichannel

## Abstract

Over 35 years ago the cell biology community was introduced to connexins as the subunit employed to assemble semicrystalline clusters of intercellular channels that had been well described morphologically as gap junctions. The decade that followed would see knowledge of the unexpectedly large 21-member human connexin family grow to reflect unique and overlapping expression patterns in all organ systems. While connexin biology initially focused on their role in constructing highly regulated intercellular channels, this was destined to change as discoveries revealed that connexin hemichannels at the cell surface had novel roles in many cell types, especially when considering connexin pathologies. Acceptance of connexins as having bifunctional channel properties was initially met with some resistance, which has given way in recent years to the premise that connexins have multifunctional properties. Depending on the connexin isoform and cell of origin, connexins have wide-ranging half-lives that vary from a couple of hours to the life expectancy of the cell. Diversity in connexin channel characteristics and molecular properties were further revealed by X-ray crystallography and single-particle cryo-EM. New avenues have seen connexins or connexin fragments playing roles in cell adhesion, tunneling nanotubes, extracellular vesicles, mitochondrial membranes, transcription regulation, and in other emerging cellular functions. These discoveries were largely linked to Cx43, which is prominent in most human organs. Here, we will review the evolution of knowledge on connexin expression in human adults and more recent evidence linking connexins to a highly diverse array of cellular functions.

As understood from the human genome project, identification of the connexin family of gap junction (GJ) proteins is likely complete with 21 members in humans (1) (Table 1). Connexin proteins have acquired a nomenclature that reflects their predicted molecular weight (*e.g.*, Cx43 = 43 kD) (2). Connexin genes are found on seven chromosomes and follow a more conventional gene nomenclature system, with GJ, followed by a Greek letter representing the subfamily and a number reflecting the order of discovery (*e.g.*, *GJA1*, gap junction alpha 1, which encodes Cx43 and was the first discovered member of the alpha subfamily) (3). Human connexin genes have now been classified into five subfamilies based on sequence homology and are denoted as GJ-A, GJ-B, GJ-C, GJ-D, or GJ-E, reflecting alpha, beta, gamma, delta, and epsilon subtypes (4, 5)(Table 1). Since 1990, there has been a virtual explosion of articles that focus on GJs, connexins, and/or Cx43 (Fig. 1). The depth of knowledge and understanding of connexin family members is variable with over 50% of published reports since 1995 focusing on Cx43 (Fig. 1). Novel discoveries revealed through interrogation of Cx43 have commonly been extrapolated to other connexin family members. Furthermore, insights into nonhuman connexins are frequently used to deduce information about their putative human orthologs. While this approach continues to provide systemic insights into the entire human connexin family, caution needs to be exercised as each connexin has many unique properties that distinguishes it from its family members. In fact, unexpected diversity even within individual connexin family members (*e.g.*, Cx43 *versus* N-terminal truncated Cx43) continues to reveal that the “same” connexins can have remarkably different functions with distinct modes of regulation.

**Table 1:** Connexin diversity and links to inherited diseases

Connexin	Gene	Organs expressed	# Of diseases associated with gene variants	High-resolution structure solved
Cx43	*GJA1*	57	6	108, 109
Cx46	*GJA3*	7	1	101, 102
Cx37	*GJA4*	18	1∗	—
Cx40	*GJA5*	27	3∗	—
Cx50	*GJA8*	3	1	101, 102
Cx59	*GJA9*	5	0	—
Cx62	*GJA10*	10	0	—
Cx32	*GJB1*	35	1	—
Cx26	*GJB2*	39	8	98–100, 103–106
Cx31	*GJB3*	10	3	—
Cx30.3	*GJB4*	9	1	—
Cx31.1	*GJB5*	8	0	—
Cx30	*GJB6*	18	4	—
Cx25	*GJB7*	10	0	—
Cx45	*GJC1*	22	1∗	—
Cx47	*GJC2*	5	3	—
Cx30.2 (Cx31.3)	*GJC3*	7	0	107
Cx36	*GJD2*	9	0	110
Cx31.9	*GJD3*	10	0	—
Cx40.1	*GJD4*	6	0	—
Cx23	*GJE1*	0	0	—

A list of the 21 connexin encoding genes and corresponding protein names. Connexin genes are typically divided into five subtypes denoted by A, B, C, D, and E, reflective of sequence homologies. The number of adult human organs where these connexins have been detected are noted as revealed by a systematic analysis of articles available on PubMed. Seventy-five organs were assessed, with 62 organs exhibiting evidence for expression of at least one connexin. Note that Cx43, Cx26, and Cx32 are the three most widely distributed connexin isoforms being found in numerous human organs. The vast majority of the 33 human diseases attributed to mutations/variants within the genes encoding twelve connexin isoforms are annotated within the Online Mendelian Inheritance in Man database, while others have been reported elsewhere. Asterisks indicate that one or more of the diseases included was identified from the Genetic Testing Registry or a PubMed search of disease-associated connexin gene variants that require further patient studies to confirm linkage. Connexin gene polymorphisms associated with disease were not included. The high-resolution structure of six connexins found within gap junctions and/or hemichannels are listed along with corresponding references.

**Figure 1:**
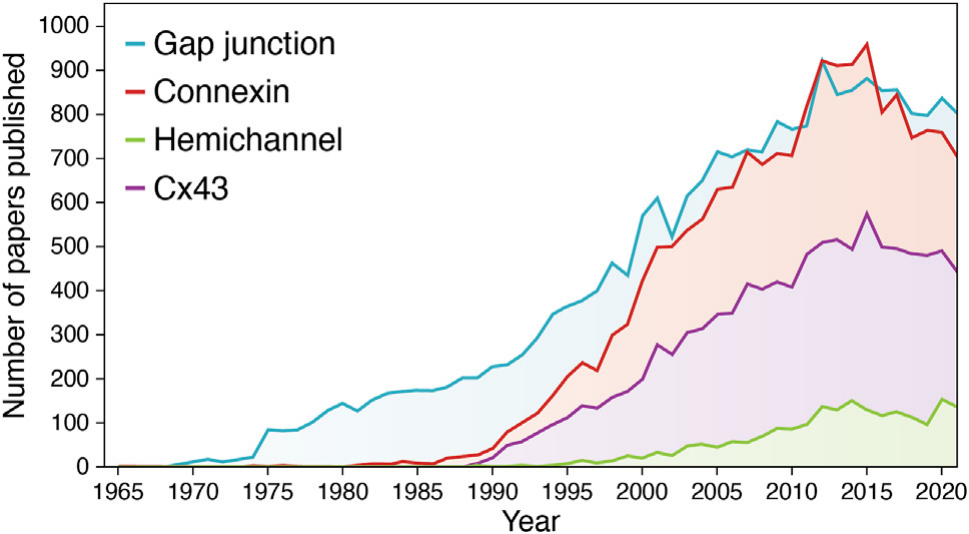
**Growth of gap junction research.** Since the discovery of gap junctions in the 1960s, connexins in the 1980s, and hemichannels in the 1990s, there has been a rapid and steady increase in knowledge on these topics as evidenced by the explosion of articles published each year. It is notable that over half of published papers in the field have focused on Cx43. Since 2015 the connexin community has seen a slight decline in articles that feature gap junctions, connexins, and Cx43, while hemichannel papers remain steady.

Canonically, in their capacity as GJ forming integral membrane proteins that span the lipid bilayer four times, connexins oligomerize into connexons (commonly referred to as hemichannels) shortly after their cotranslational insertion into the endoplasmic reticulum membrane (6, 7, 8) (Fig. 2, *A* and *C*). In at least a couple of exceptions, this oligomerization process may be delayed until they reach the Golgi apparatus (9, 10). Transport vesicles actively and constitutively carry connexons to the cell surface, where they dock with compatible connexons from a juxtaposed cell to form intercellular channels that allow for gap junctional intercellular communication (GJIC) (11). These intercellular channels quickly cluster into tightly packed arrays that have historically been termed GJs, structures that are interchangeably referred to as GJ plaques (12) (Fig. 2*C*). A single GJ can easily contain hundreds to thousands of channels allowing for a massive site of regulated intercellular exchange of ions and potentially thousands of members of the metabolome that are less than 1 kD in size (13, 14, 15, 16)(Fig. 2, *A*–*C*). While it is well known that small molecules can pass through GJ channels, direct evidence of which molecules do is limited to less than two dozen, including ATP, cAMP, IP_3_, GSH, nucleotides, ions, and at least some amino acids (17, 18) (Fig. 3). It is further unclear which of these transjunctional molecules can pass through GJ channel conduits composed of different connexin constituents. Given the brevity of the present article, this aspect of channel complexity is reviewed in more detail elsewhere (17, 18, 19). GJ-mediated molecular exchange has fundamental importance in healthy cell signaling and can lead to pathologies if perturbed by loss- or gain-of function connexin gene mutations or dysregulation of connexin expression (20, 21, 22).

**Figure 2:**
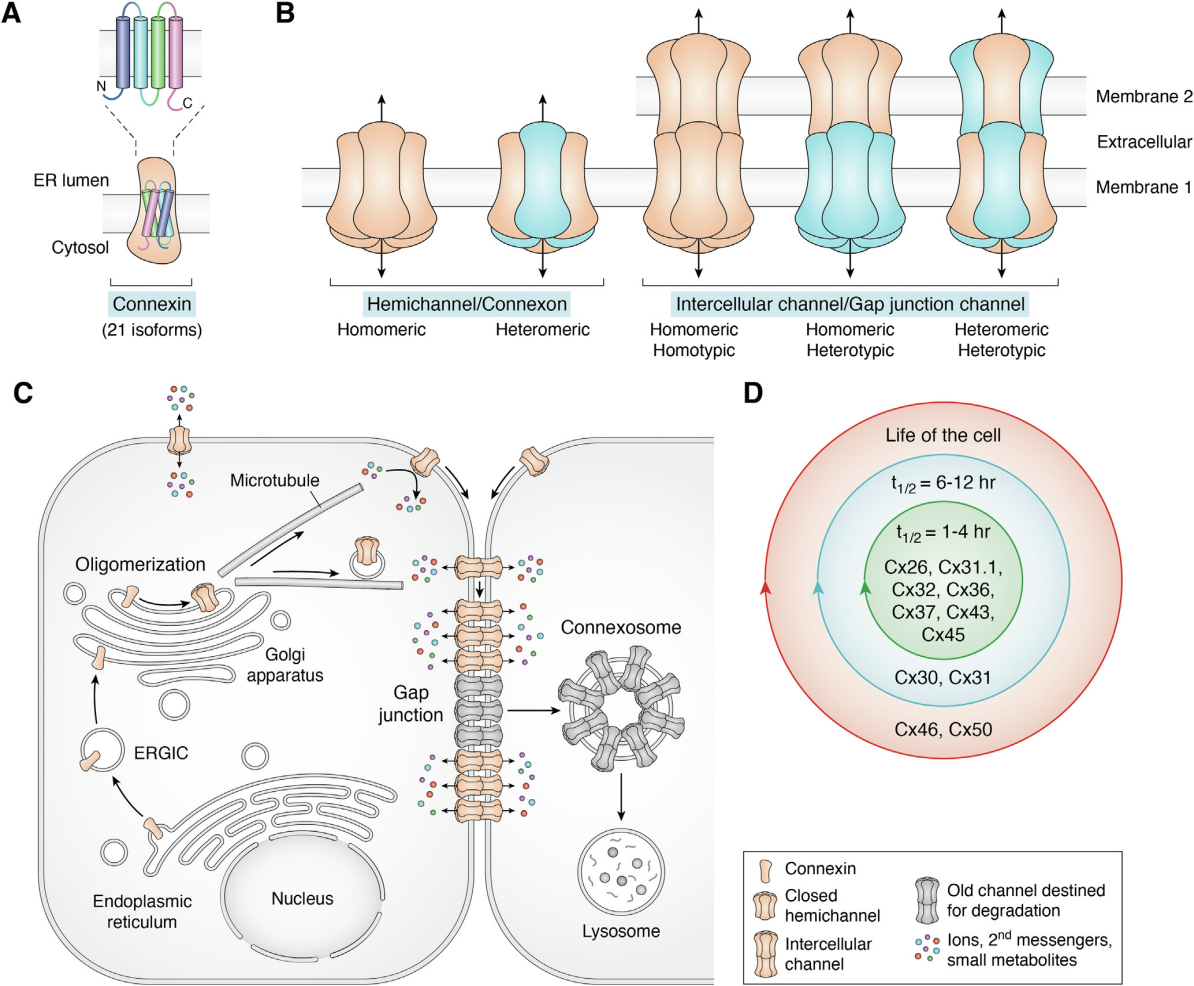
**Organization and life cycle of connexins.***A*, connexins are polytopic integral membrane proteins that pass through the lipid bilayer four times during their insertion into the endoplasmic reticulum (ER) membrane. *B*, properly folded connexins oligomerize into hexamers termed connexons that can function as hemichannels in single membranes. Connexons can be homomeric or engage two or more connexin isoforms and be heteromeric. At the cell surface connexons assemble into a wide combination of homomeric, heteromeric, homotypic, or heterotypic channel arrangements. *C*, the typical life cycle of connexins as elucidated largely on the interrogation of Cx43. Connexins undergo biosynthesis and cotranslational import into the endoplasmic reticulum prior to being transported through the ER-Golgi intermediate compartment (ERGIC) to the Golgi apparatus. After oligomerization, connexons are delivered to the cell surface, where they may function as hemichannels prior to docking with adjacent connexons to form gap junction (GJ) channels that are suitable for the direct intercellular exchange of metabolites. GJ channels cluster into gap junction plaques with newer channels being found at the edges of the plaques. GJs and GJ fragments may proceed to internalize into double-membrane structures called connexosomes that proceed to lysosomes for degradation. *D*, diversity in the half-life of connexins. The majority of interrogated connexins have been found to have a relatively short half-life of 1 to 4 h with others having longer relative half-lives depending on the connexin isoform and the cellular context in which they are found with some connexins (*e.g.*, Cx46, Cx50) existing for the entire life of the cell as seen in anuclear lens fiber cells.

**Figure 3:**
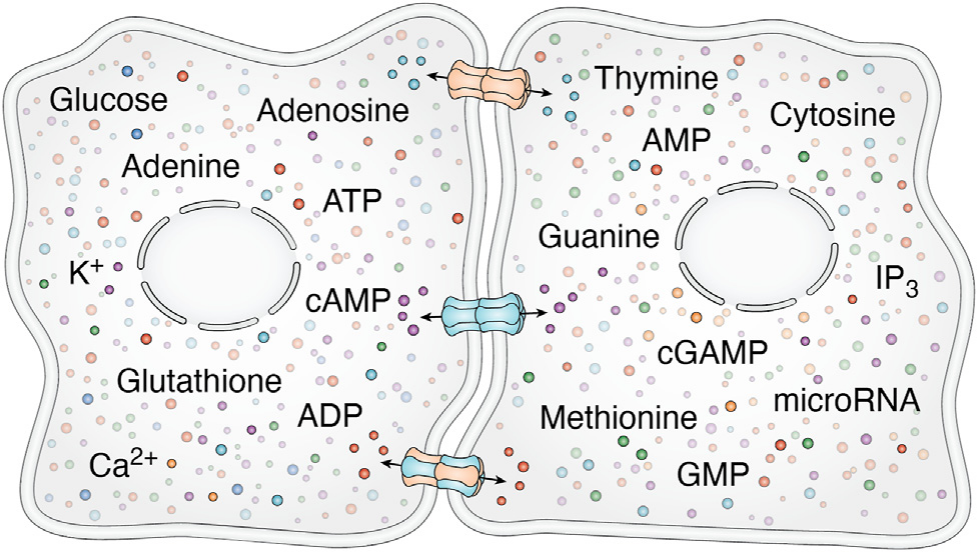
**Complexity of GJIC.** GJIC may involve the potential passage of literally thousands of members of the metabolome through homotypic or heterotypic channels. Selective passage of ions, signaling molecules, and metabolites is highly dependent on the connexin isoforms (depicted as *red/blue subunits*) used to build gap junction channels. Identification of transjunctional molecules is difficult, so not surprising, direct evidence for the passage of metabolites exists for only a small subset of molecules found within the metabolome. Part of the figure was generated using BioRender. For a movie animation of this figure see: https://www.schulich.uwo.ca/lairdlab/img/cell-animation-small.mp4. GJIC, gap junctional intercellular communication.

## Connexins as hemichannels

Within a decade of connexins being identified as the proteins that lined the pore of GJ channels, they were proposed to have a second function in serving as single membrane channels at the plasma membrane (hemichannels) (23). Early on this concept met considerable resistance in the GJ community as cell surface connexons were proposed to be transient structures that remained closed as they quickly proceeded to dock with connexons from an adjacent cell to form GJ channels. Opposition to connexins acting in a hemichannel context could also stem from an evolutionary perspective as this might be considered a “step-down” function to connexins acting as unique direct cell–cell channel conduits. It is notable that in addition to connexins, several membrane transport proteins and channels are capable of actively or passively moving small regulator molecules across the plasma membrane of human cells (*e.g.*, ion selective channels, pannexins, calcium homeostasis modulator) (24, 25). Hemichannel redundancy arguments also pointed to the notion that connexins are not unique in forming large-pore channels that allow molecules larger than ions to pass (25). However, in vertebrates, other than connexins, there is no other protein family that has acquired the evolutionary advantage of direct and regulated intercellular exchange of signaling molecules barring the exception that pannexin 1 potentially acquires this capacity in rare situations (26, 27). Nevertheless, overwhelming experimental evidence now points to cell surface connexin hemichannels having key roles in normal chordate physiology, pathology, and tissue injury, all of which are reviewed in detail elsewhere (28, 29, 30, 31).

The regulated opening of cell surface hemichannels provides a conduit for the release of many small molecules (*e.g.*, GSH, glutamate, others) from the cytosol to the extracellular milieu with the best characterized of these being ATP (32). Hemichannels not only enable bidirectional diffusion of small molecules such as a plethora of informative dyes (*e.g.*, ethidium bromide, YO-PRO) (33, 34) that are used for assessing hemichannel function, but also biologically relevant molecules (*e.g.*, calcium) (35). When considering small molecule exchange between the cytosol and extracellular milieu, it is important to be cognizant that these two microenvironments have substantially different molecular component compositions that are necessary to ensure a healthy local environment for cell survival. While it is not our intent here to cover the breadth of evidence that supports a key role for connexin hemichannels, we would be remiss not to give a couple of examples. The first is in the lens where Cx46 hemichannels play a critical role in maintaining lens homeostasis (36, 37, 38). The precise roles that Cx43, Cx46, or Cx50 hemichannels play in normal human lens remains somewhat elusive. However, models have been proposed where mechanosensitive hemichannels accommodate a steady-state fluid equilibrium and pathways for the influx of calcium and sodium, and the efflux of potassium (37, 38) required for normal lens function and maintenance of transparency. Amplified hemichannel activity, as seen for some diseased-linked Cx46 and Cx50 gene mutations (39), may lead to ATP and GSH leaking from lens cells harboring these mutants culminating in cytotoxicity and/or cataracts. In another example, mechanosensitive hemichannels are found in bone osteocytes, where they appear to play a role in bone remodeling and plasticity through their regulated release of ATP, NAD^+^, and prostaglandin E_2_ (40). While it is expected that few hemichannels are open at steady state, they can be regulated to open in response to changes in the microenvironment such as calcium reduction. In this context, connexin hemichannels would be expected to react to oxidative stress as a molecular means to protect osteocytes from cell death (41, 42).

Given the recognition that hyperactive or leaky hemichannels are linked to pathologies in the brain, eyes, bone, skin, lens, and other organs (43, 44), they are now being considered as viable targets for therapeutics (21, 45, 46, 47, 48). Of note, a recent study using a newly developed hemichannel blocker D4 was found to greatly attenuate seizures in a mouse model of temporal lobe epilepsy by reducing neuroinflammation (49). Another preclinical study found that an antibody directed to Cx26 potently improved the skin pathology found in a mouse model of keratitis ichthyosis deafness syndrome (46). Preclinical studies using inhibitors of hemichannels continue to arise that show promise in having efficacy in pathologies and injury scenarios. However, caution needs to be exercised in using pharmacological inhibitors that are known to have a broad spectrum of effects on diseased and healthy cells.

## Diversity in connexin channel composition

Complexity in GJ channel formation begins at the expression level as most human cells express two or more connexin isoforms, resulting in the potential for a mixed array of connexons that can be either homomeric or heteromeric (50) (Fig. 2*B*). Connexins are selective in which connexin isoforms they can co-oligomerize with, thus attenuating the potential number of connexon subtypes that can form (50, 51, 52, 53) (Fig. 2*B*). At the cell surface, connexons are also selective in their docking capacity with connexons from an adjacent cell, establishing a further layer of homotypic and heterotypic intercellular communication possibilities (54, 55, 56, 57) (Fig. 2*B*). Finally, GJs can form across different cell types, including cells that are at progressive stages of differentiation such as that found in the epidermis, leading to a wealth of homocellular and heterocellular intercellular communication networks (58, 59, 60). In addition to GJIC being regulated by channel composition, channels are further governed by gating mechanisms in response to changes in pH, voltage, ion concentrations, protein interactions, phosphorylation states, and other mechanisms (52, 61, 62). GJ channel gating mechanisms have been discussed extensively elsewhere (63, 64, 65, 66, 67, 68). Given the extensive number of family members, oligomeric combinations, and regulatory mechanisms, it is not surprising that from an evolutionary standpoint connexins are well positioned to support a multitude of essential cellular functions.

## Diversity in connexin life expectancy

Evidence has emerged over several decades that points to connexins exhibiting three distinct turnover or half-life classifications reflecting not only the connexin isoform expressed but also the cellular context where it is found (Fig. 2*D*). The first of these classifications was surprising when pulse-chase studies in the liver predicted GJ turnover might be fast (69). This was soon confirmed to be 5 h (70) and later connexin turnover in hepatocytes was found to be 2.5 to 3.0 h (71). Complementing the notion that Cx26 and Cx32 found in the liver exhibit a short half-life (72), Cx37, Cx43, and Cx45 were all later found to share this rapid half-life of only 1.3 to 3.0 h in primary cardiomyocytes, a cardiomyocyte cell line, and/or heart tissue (73, 74, 75, 76). Cx36, which is commonly found in long-lived neurons, was also found to belong to this classification with a reported half-life of ∼3 h (77). Collectively with Cx31.1 (78), no less than a half-dozen connexins have been reported to share this feature of transitioning through a complex life cycle at an unprecedented rate for an integral membrane protein, which would see organs like the heart essentially renew all of its GJs daily (79). The second class of connexins represented by Cx31 (t_1/2_ ∼6 h) (80, 81) and Cx30 (t_1/2_ >12 h) (82) have longer relative half-lives than others (*e.g.*, Cx43) to suggest that they are potentially subject to different modes of regulation during their life cycle. Here, it is important to note that Cx30 and Cx31 degradation was assessed in the absence of protein synthesis (81, 82). The third classification and longest lived connexins, are Cx46 and Cx50 when found in anuclear lens fiber cells that exist for the entire human lifespan (83), albeit the functionality of these connexins greatly deteriorates during aging (84). Lens-specific cellular and/or microenvironmental cues appear to be the driving force for the longevity of at least Cx46 as it exhibits a short half-life of only a few hours when expressed in HeLa cells (85). In at least a few cases, changes to connexin turnover have been implicated in disease pointing to the importance of highly regulated connexin turnover (86, 87, 88, 89). The field awaits the classification of the half-life properties of the remaining connexins, but it might be reasonable to predict they will be relatively short unless found in highly specialized cells.

## Diversity in the distribution of connexin family members in humans

**Figure 4:**
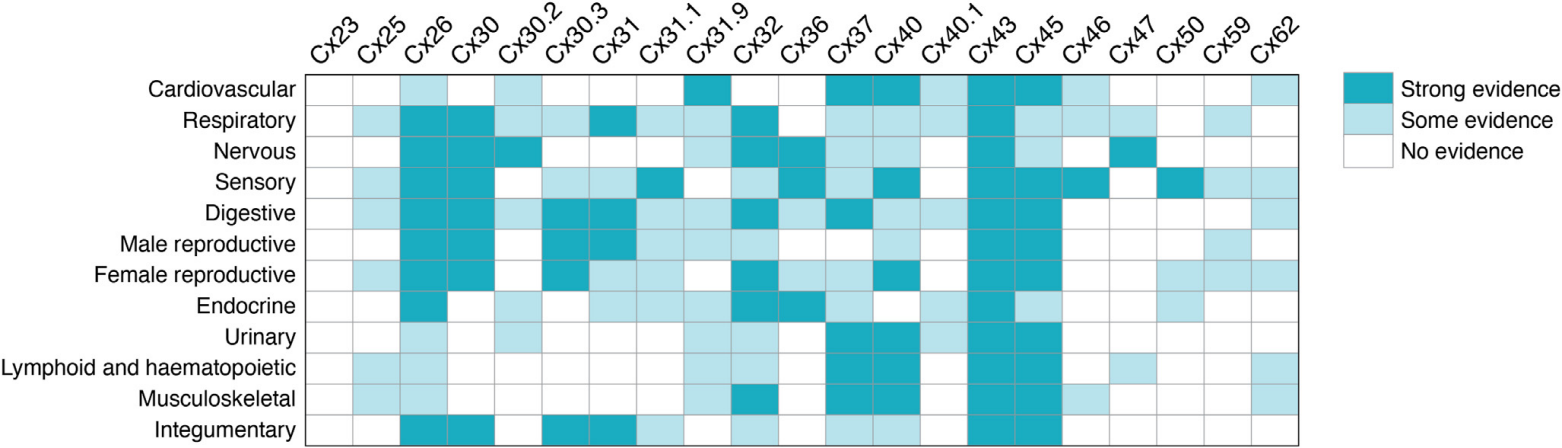
**Expression of human connexin isoforms in all 12 adult body systems.** Heatmap depiction of the expression of 21 human connexin isoforms in all adult human body systems as revealed by a systematic analysis of articles available on PubMed. Strong evidence for the expression of a connexin reflects situations where a connexin was observed by multiple laboratories using various detection approaches. Some evidence of connexin expression reflects situations where there is limited evidence of the connexin expression, and no evidence of connexin expression denotes situations where the connexin was either not investigated or negative data was obtained. Note that Cx43 is found in all systems, while other connexins are variably expressed across the adult human anatomy. This data compilation should be seen as a guide as additional insights into where connexins are expressed in adult human cells and tissues continue to emerge and be confirmed at the mRNA and protein levels.

**Figure 5:**
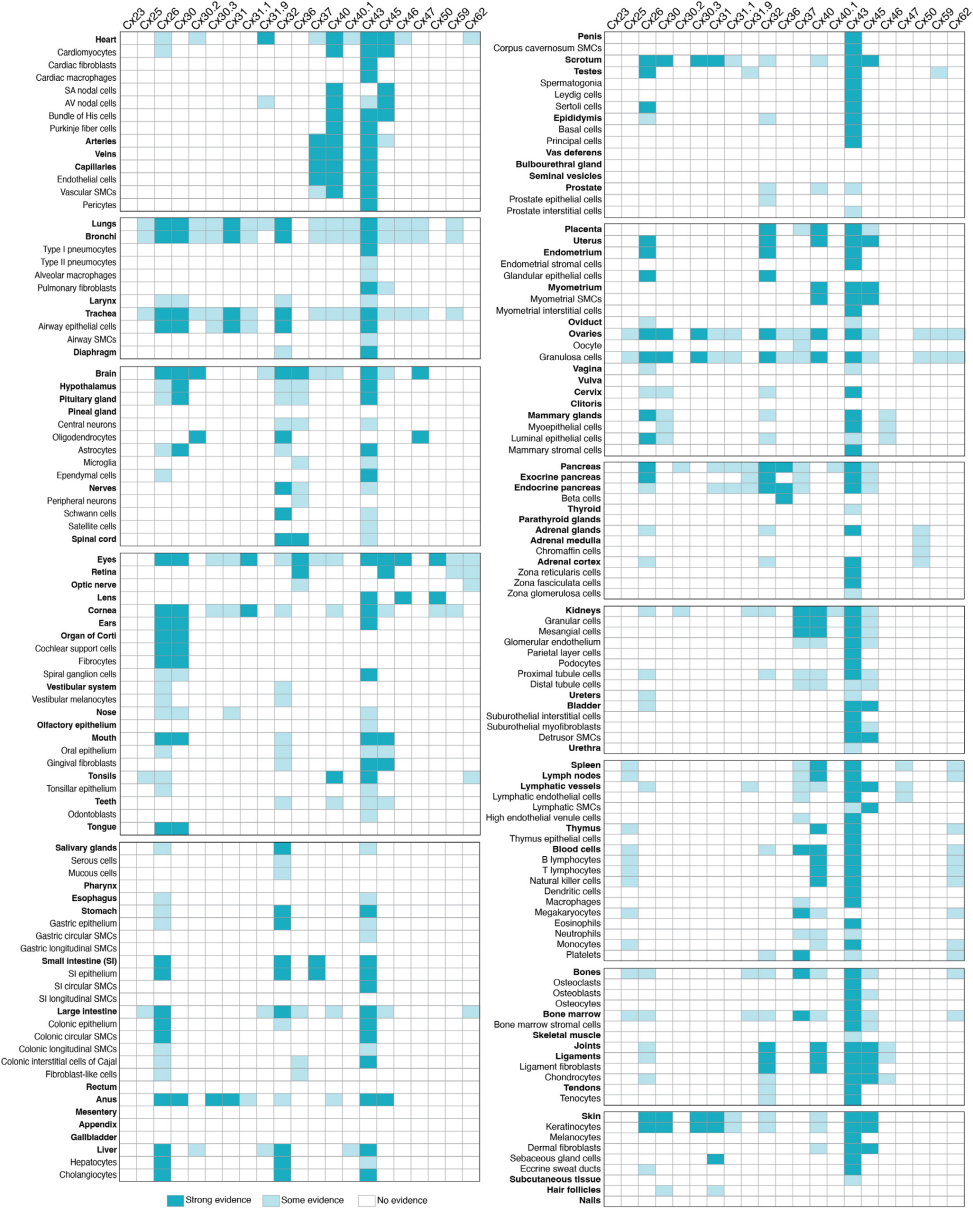
**Heatmap depiction of connexin isoform expression in adult human organs, tissues, and cells as revealed *in situ* or primary cell culture.** Note the near ubiquitous distribution of Cx43, the broad distribution of Cx26 and Cx32, and the highly restricted distribution of Cx23, Cx25, Cx59, and Cx62. Given that high-avidity antibodies do not exist for all 21 connexin isoforms and the fact that not all isoforms have been intensely investigated, it is likely that connexin isoforms will be detected in more adult human organs, tissues, and cell types in the future. Heatmap was generated as described in Figure 4. Organs/tissues are noted in *bold*. Compartmentalization of the list represents the 12 body systems. SMC, smooth muscle cell.

Diversity in connexin gene expression is found in all 12 human body systems, dozens of organs, and numerous cell types (Figs. 4 and 5). To investigate connexin diversity in humans, we undertook an analysis of over 200 articles found within the PubMed database that collectively referred to over 110 human cell types (Figs. 4 and 5, Table S1). Our approach was to consider only healthy adult tissues and primary cells avoiding complexities associated with prenatal development or cells that had entered a disease state. For our analysis, strong evidence of connexin expression reflected situations where both connexin encoding mRNA and connexin proteins were detected using multiple techniques on clinically obtained samples and/or primary cells. Some evidence of connexin expression reflected a variety of situations that included when a connexin isoform was detected only at the transcription level, or by only one methodology, and/or where only a weak signal was noted in a single study. No evidence of connexin expression was reserved for either negative finding or where no studies were identified. We could not confidently refine this last category as it was unclear if high-quality reagents were used in all studies where connexins were not detected. This analysis does not account for online annotated microarray, mRNA, and RNA-seq data bases (*e.g.*, gene expression omnibus (www.ncbi.nlm.nih/geo/), which may not necessarily predict protein expression and require a much deeper dive bioinformatics approach to analyze. We also did not include datasets from the Human Protein Atlas (https://www.proteinatlas.org/) as high-fidelity antibodies do not exist for many of the 21 connexin family members at the present time, which can lead to erroneous outcomes. Lastly, evidence of a connexin isoform expression in normal cells and tissues of rodents or other species was not extrapolated to the human heatmap displays (Figs. 4 and 5), since some connexin expression patterns differ amongst species. Acknowledging these limitations, we were able to make some general observations on the connexin expression profile in human cells and tissues as we consider the relationship between the genome and phenome.

First, Cx43 is expressed in all 12 major human body systems, in nearly all organs and over 90% of cell types identified within the studies analyzed (Figs. 4 and 5). This ubiquitous expression pattern points to essential roles for Cx43 in human cell differentiation and homeostasis. In later sections, we will discuss how Cx43 has become the focal point for many noncanonical connexin studies linked to its subcellular distribution beyond being found in GJs. Cx26 and Cx32 were identified in most body systems, but their expression was variable and more restrictive in adult tissues. Collectively, these three connexins have received the most attention in the connexin field due to their broad distribution and the availability of high-affinity antibodies to track their temporal and spatial localization in cells and tissues. Consequently, topological, structural, and functional discoveries and insights into these connexins have been widely extrapolated to other connexin family members. However, caution needs to be exercised in this approach as the unique features of individual connexin family members may override connexin generalizations.

It is notable that a strikingly large number of connexin isoforms have been reported in some organs like the brain, eyes, ovaries, pancreas, kidneys, and skin (Fig. 5). These findings may reflect the complexity of cell types and cell specializations found within these organs and the need for highly refined GJIC. All but six connexin isoforms have been reported in one or more tissues of the eye reflecting the sophistication of the cells that constitute this sensory organ (90) (Fig. 5). In the skin, the continual renewal of the epidermis requires keratinocytes to undergo gradual and complex states of differentiation leading to cell death that is accompanied by the temporal and spatial expression of at least nine connexin isoforms (22) (Fig. 5). Surprisingly, the field is waiting for insights into which connexins are expressed in the human appendix and gallbladder, as they remain remarkably understudied considering the expected availability of surgically excised tissue.

As an extreme example of the uniqueness of connexin family members, unlike most connexins that are encoded by one exon, Cx23 is encoded by three exons (91). However, as a protein, Cx23 has not been detected in human adults though it has been reported in the developing fetal cochlea (92). Depending on the splicing pattern, a stop codon may be engaged at the start of the third exon or, alternatively, a different splice acceptor site may become available and yield a human Cx23 protein that is four amino acids shorter than the mouse ortholog (91). Both the mouse and human Cx23 gene encode four extracellular facing cysteines instead of the six found in all other connexins. This difference may account for mouse Cx23 being assembled into functional hemichannels but not GJ channels (91), although zebrafish Cx23 appears to form functional GJs (93). Given these findings it is possible that Cx23 is not expressed in adult humans as a GJ channel forming protein. Knowledge of human Cx25 is also elusive but some evidence has been reported in the respiratory, lymphoid, hematopoietic, and female reproduction systems (94, 95, 96). Given that Cx25 has no mouse ortholog, it has remained underinvestigated, but its expression appears to be linked to developmental processes or diseased scenarios such as in leukemic cells (95). Cx59 also has no mouse ortholog and has been reported to be found in the retina along with Cx62 (97). Overall, evidence supports the notion that Cx25, Cx59, and Cx62 are all limitedly expressed in humans and may be reserved for specialized cells, where GJIC needs are unique and not fulfilled by other coexpressed connexins. The remaining 14 connexin isoforms (Table 1, Figs. 4 and 5) are variably, but also limitedly, expressed amongst all organs, tissues, and cells. It stands to reason that cell specialization dictates which connexin genes need to be expressed to accomplish cell functions, which extend beyond contiguous cells within multicellular tissues to cells that are freely circulating in bodily fluids (*e.g.*, lymphocytes and hematopoietic cells).

## High-resolution structural analysis reveals connexin channel diversity

Solving the high-resolution structure of integral membrane proteins took a quantum leap forward over the last couple of decades and transformed the understanding of connexin channels. Notably, 3.5 Å crystallographic structures of Cx26 (a beta connexin) paved the way for structure interrogation of GJ channels (98) (Table 1). Tsukihara’s team used high-resolution structures to confirm the tetraspanning nature of a single Cx26 molecule and the hexameric organization of protomers into a hemichannel with a well-defined pore (98). A commonly found Cx26 mutant (M34A) was also solved and revealed that this mutation tended to drive Cx26 channels into a closed state, a process that involved the N termini (99, 100). Single-particle cryo-EM revolutionized the molecular understanding of connexin channels as detailed analysis of lens GJ channels composed of Cx46/Cx50 (alpha connexins) in their native state revealed these connexins acquired a more stable open state (101). Pushing the connexin structure resolution obtained from cryo-EM to an unprecedented 1.9 Å showed that when Cx46/Cx50 intercellular channels were assessed in a dual lipid nanodisc system, water was localized to the pore regions and lipids were found to stabilize the structure (102). Revisiting Cx26 structure, X-ray crystallography of human Cx26 GJ channels unexpectedly showed that bound calcium did not cause a major structure change that would lead to pore occlusion, pointing to electrostatic changes as being responsible for calcium-mediated gating (103). Later, cryo-EM analysis of Cx26 channel structure at physiological pH and pH 6.4 revealed open and closed conformational states that were linked to the positioning of the amino terminus (104). Moreover, a N176Y mutation in Cx26 led to hemichannels acquiring an open state in lipid bilayer nanodiscs (105). Further analysis of Cx26 *via* cryo-EM disclosed that CO_2_ partial pressure changed the pore-lining helices to reduce the aperture of the GJ channel pore (106). Some of the highest resolution cryo-EM in connexin biology showed that Cx31.3 (a gamma connexin) hemichannels had a ∼8 Å pore suitable for selective permeability to chloride ions (107), pointing to how different connexins may establish channel selectivity. Most recently the high-resolution structure of Cx43 (an alpha connexin) was solved by cryo-EM, and it was found to exist in four dynamic states that resulted in a wide range of pore sizes including a closed state (108, 109). In the same year, human Cx36 channels, which are prevalent in neurons, were interrogated by cryo-EM and shown to exist in a closed state where lipids block the pore, suggesting lipids play an important role in channel gating (110). There is no doubt that as high-resolution structures of further connexin GJ channels and hemichannels are solved, possibly including the structures of the cytoplasmic loop and carboxy-terminal domains, discoveries will continue to reveal that different connexin family members have unique structural properties that point to multiple mechanisms of channel regulation.

## Noncanonical roles of connexins and connexins fragments

**Figure 6:**
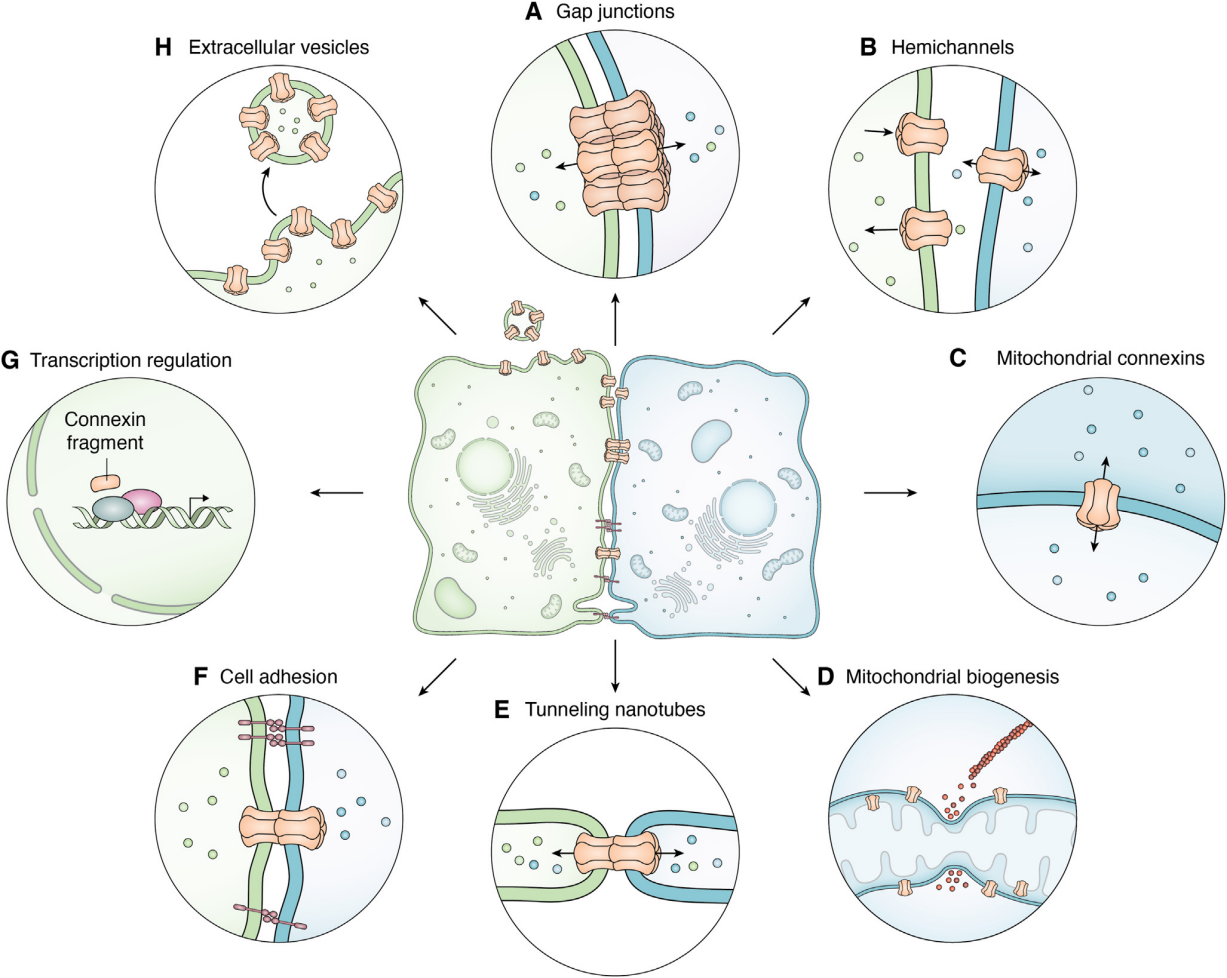
**Schematic depiction of the functional diversity of connexins.** Once thought to be solely linked to GJIC (*A*) and later to hemichannel functions within the plasma membrane (*B*), evidence now supports connexins or connexin fragments as having roles in the mitochondria (*C* and *D*), tunneling nanotubules (*E*), cell adhesion (*F*), transcription (*G*), and within extracellular vesicle membranes (*H*). Other emerging cellular roles for Cx43 are not shown.

If one takes the approach of classifying both GJIC and cell surface hemichannel functions as representing canonical roles for connexins, an ever-growing list of noncanonical roles has emerged (Fig. 6). Historically, the engagement of Cx32 in Schwann cells at Schmidt–Lanterman clefts, to allow for intracellular transport of metabolites across myelin sheath, established the principle that connexins may have noncanonical roles in at least highly specialized cells (111, 112). Noncanonical roles may still involve connexins when organized into GJ channels (*e.g.*, tunneling nanotubes), but often these roles involve connexins acting as single membrane channels (*e.g.*, mitochondrial membranes) or even as N-terminal truncated fragments (*e.g.*, GJA1-20k). Here, we will give a brief overview of several noncanonical roles that have recently been elucidated while still further roles for connexins, such as in invadopodia (113) and phagocytosis, continue to be interrogated (114, 115, 116).

### Amino-terminal truncated fragments of Cx43

While connexin expression is commonly regulated through cap-dependent translation, a growing body of evidence suggests some connexin isoforms contain internal ribosomal entry sites and can subsequently undergo cap-independent translation (117, 118). Generally, cap-independent translation is enhanced in pathological conditions, where cap-dependent translation is impaired such as in the case of starvation, cellular stress, apoptosis, and disease (118). While the presence of internal ribosomal entry sites elements have been documented for Cx43, Cx32, Cx26, and zebrafish Cx55.5, the internal translation of Cx43 remains the most elucidated (118, 119, 120, 121, 122). With seven in-frame methionine codons present in the Cx43 coding sequence, several N-terminal truncated Cx43 isoforms have been proposed to exist (GJA1-32k, GJA1-29k, GJA1-26k, GJA1-20k, GJA1-11k, and GJA1-7k), but focus has been on the most abundant GJA1-20k isoform (produced from translation initiation at M213) found in heart cells (123). It has been reported that the GJA1-20k fragment self-regulates full-length Cx43 by promoting oligomerization and stabilizing the actin cytoskeleton, which consequently facilitates connexon trafficking (124, 125). Interestingly, in a mouse model where the methionine corresponding to the start-site of the GJA1-20k fragment was mutated to a leucine, preventing the biosynthesis of the GJA1-20k fragment, homozygous mutant mice die young from apparent sudden cardiac death (126). These findings provide evidence that that GJA1-20k contributes to the maintenance of normal physiological processes and the localization of full-length Cx43.

In addition to GJA1-20k functioning as a self-regulator, it has also been localized to the nucleus where it may elicit additional channel-independent functions (127). Here, GJA1-20k was proposed to be recruited to the nucleus by basic transcription factor 3 where it forms a complex with polymerase II to regulate N-cadherin transcription (127) directly implicating a connexin in regulating cellular adhesion at the transcriptional level. Nuclear localization of Cx43 or possibly a C-terminal Cx43 fragment was seen in Wnt-5A– and Wnt-9B–treated cells, whereas TNFα decreased Cx43 nuclear localization prompting the speculation that nuclear Cx43 may be involved in various signaling cascades (128, 129). Another study suggests Cx43 is translocated to the nucleus during late G1 phase of the cell cycle by A-kinase anchoring protein 95 (130). Further, a GJA1-11k fragment has been recently reported to enter the nucleus and regulate cell growth and cell cycle progression (131, 132). Despite these findings, a nuclear localization signal for Cx43 has yet to be identified (131, 132) but may not be needed given its small size. However, it remains elusive whether the connexin fragments are key transcriptional regulators or accessories that support gene transcription systems.

### Mitochondrial connexins and connexin fragments

While connexins are canonically thought of as proteins that form cell surface hemichannels and GJs, there is increasing evidence that at least some connexins localize and function within the mitochondria. This notion of so called “mitochondrial connexins” was first proposed over two decades ago (133). Since then, mitochondrial connexins have been extensively investigated in a variety of cell types with dozens of studies focusing on mitochondrial Cx43 in cardiomyocytes from normal and diseased hearts as recently reviewed in detail (134, 135, 136). Cx32 and Cx26 have also been reported in the inner mitochondrial membranes of hepatocytes (137), while Cx40 was found in mitochondrial membranes of endothelial cells where it has been proposed to regulate calcium homeostasis (138). Electron microscopy imaging data has identified sites where internalized GJs are in contact with mitochondria, suggesting that they may structurally cross-talk for reasons that remain unclear (139). It is also unclear if the remaining connexin family members ever acquire residency within the mitochondria as the vast majority of these have not been fully investigated.

In cardiomyocytes, Cx43 localization is well described primarily within the intercalated discs as GJ channels and to a lesser extent in the sarcolemma as hemichannels. Evidence has also emerged that Cx43 localizes to a subpopulation of mitochondria that reside just below the sarcolemma with little Cx43 being found in the mitochondria between the myofibrils (140). It is not clear if a similar dual population of Cx43-enriched and Cx43-deficient mitochondria exists in skeletal muscle myocytes or in other cell types. Given that Cx43 is ubiquitous to at least half of the cell types in the human body, it also remains unclear where, when, and why mitochondrial Cx43 has only been reported in a subset of cell types that includes myocytes, endothelial cells, hepatocytes, and adipocytes. This may be simply the consequence of a lack of research intensity into mitochondrial connexins or it may reflect cell type specializations that are not ubiquitous.

While it has become more widely accepted that Cx43 serves a noncanonical role within the mitochondria it remains to be clarified if this role occurs as a full-length protein in its monomeric form, as a truncated polypeptide, or when organized into a hemichannel (141). The role of mitochondrial Cx43 has been linked to both the inner membrane (142) and outer mitochondrial membranes (136, 143), where it might serve distinctly different functions. As a full-length integral membrane protein, it has been proposed that the translocase of the outer membrane and heat shock protein 90 participate in Cx43 import across the outer mitochondrial membrane as it seeks to insert into the inner mitochondrial membranes (142). Details related to how this occurs are still lacking as is knowledge concerning where and when mitochondrial Cx43 undergoes oligomerization into a putative hemichannel. Likewise, mechanisms for how Cx26, Cx32, or Cx40 may enter mitochondrial membranes remain ill-defined. Functional mitochondrial connexin–based channels have been reported that are capable of dye uptake potentially allowing them to serve as regulatable conduits for potassium and calcium influx (141, 144). Dye uptake experiments further suggest that Cx43 forms functional mitochondrial hemichannels with enhanced dye uptake occurring in response to increased delivery of Cx43 to the mitochondria due to oxidative stress (145). Furthermore, Cx43 has been suggested to regulate mitochondrial calcium uptake (*via* interactions with the mitochondrial calcium uniporter) (146), mitochondrial respiration and ATP production (145, 147), reactive oxygen species production (145, 148), and the mitochondrial membrane potential (145). In keeping with these findings, Cx40 ablation and overexpression experiments in endothelial cells indicate that Cx40 regulates mitochondrial calcium homeostasis and reactive oxygen species formation (138). However, it is not clear whether these findings were mediated by mitochondrial Cx40 hemichannels or GJs (138).

The GJA1-20k fragment has also been reported to associate with mitochondria, where it has been proposed to have multiple roles ranging from assisting in microtubule-dependent mitochondrial transport (149) to promoting mitochondrial biogenesis and cardiac protection against ischemia/reperfusion injury (150) to providing protection against mitochondrial fission (151). For example, overexpressing GJA1-20k in an angiotensin II–induced cardiomyocyte hypertrophy model modulated mitochondrial membrane potential and respiration while lowering mitochondrial superoxide production suggesting a potential role in regulating mitochondrial function (152). All these characteristics attest to a role for GJA1-20k in regulating mitochondrial dynamics, while this polypeptide is potentially associated with mitochondrial membranes (134, 136). Overall, it is now well documented that a subpopulation of connexins localize within the mitochondria and impact mitochondrial function. However, the field awaits further clarity on how connexins or connexins fragments are targeted to mitochondria, where they appear to serve key noncanonical roles.

### Connexins in cell adhesion

The concept and proposition that connexins contribute to the nexus of cell–cell adhesion has existed for decades (7, 153, 154). This concept centers around the myriad of evidence that GJs work together with structural junctions known as desmosomes, adherens junctions, and tight junctions to establish a robust nexus of cell adhesion in developing and adult multicellular tissues and organs (155). For example, the Cx43 intracellular interactome shares members with the interactomes of claudins, cadherins, and desmosomal proteins (156, 157, 158, 159), suggesting that the upregulation or downregulation of at least some connexin members would have implications on the availability of binding proteins engaged in structural junction homeostasis. Recently, the second extracellular loop domain of Cx50 was found to be implicated in driving cell–cell adhesion through the regulated expression of adhesion molecules with direct implications in lens cell differentiation (160, 161). GJs composed of Cx26 and/or Cx43 have been reported to establish adhesive sites at locations between radial fibers and neurons where they serve to guide neuronal migration (162). However, at least one case exists where increased Cx26 function reduces N-cadherin expression and cell adhesion (163), pointing to a complex relationship between GJs and structural junctions. Of note, hydrogen bonds and salt bridges have been reported to exist between docked Cx26 hemichannels, which can considerably contribute to overall cell adhesion (98). As an approximation, the cryo-EM solved structure of Cx26 (PDB: 7QER) predicts that up to 96 hydrogen bonds (PDBePISA analysis of the 7QER structure) are involved in docking of two Cx26 hexamers into a single intercellular channel (106). Assuming the strength of hydrogen bonds to be at least 0.5 kcal/mol for proteins in solution (164), then each channel contributes an adhesion strength of at least 48 kcal/mol not including adhesive forces from other noncovalent interactions. When we consider that GJ consists of hundreds or more channels (7, 165, 166), then the adhesion strength of an individual GJ might easily exceed 5000 kcal/mol, providing further evidence that GJs may meaningfully contribute to cell adhesion. Nevertheless, their direct contribution to adhesion is limited by the fact that GJs are not strongly anchored to cytoskeletal elements like structural junctions (*e.g.*, adherens junctions, desmosomes, tight junctions).

### Connexins as mediators of long-range and specialized intercellular communication

Although connexins are known to facilitate the direct intercellular exchange of ions and metabolites *via* GJIC, almost two decades ago actin-containing membrane protrusions called tunneling nanotubules (TNTs) emerged as specialized sites used to establish distant intercellular communication (167). Originally defined as open-ended protrusions with membrane continuity, evidence has emerged that a subset of TNTs contains close-ended protrusions coupled *via* connexin intercellular channels (168, 169, 170, 171). However, the relationship between connexins and TNTs is poorly characterized at present with most reports investigating the role of Cx43 *in vitro* with few studies exploring *in vivo* models and the engagement of other connexins. Cx43 appears to play a role in TNT formation and their morphological properties. As an example, GJIC-deficient ovarian cancer cells developed shorter TNTs than their GJIC-competent controls (172). In keeping with these observations, the deletion, knockdown, or inhibition of Cx43 has been shown to significantly reduce the length and abundance of TNTs (173, 174, 175, 176). Since most human cell types express at least two connexin isoforms, it is interesting to consider how other connexins dynamically and differentially contribute to TNT physiology. In HeLa cells, it was found that the overexpression of Cx36 notably promoted the formation of TNTs in comparison to Cx43-, Cx45-, and Cx47-expressing cells, where TNTs were less frequently found indicating differential roles for connexin isoforms in modulating TNT formation (177). As a result, it is important to examine how distinct connexin isoforms uniquely contribute to specialized intercellular communication *via* TNTs *in vivo*.

In recent years, the characterization and classification of extracellular vesicles (EVs) has advanced with the identification of many subtypes that may have direct importance in disease diagnostics and in the delivery of therapeutics (178). While EVs can transport physiological and pathophysiological cargo such as proteins, RNA, and DNA to recipient cells, the unique mechanisms governing their targeting and release remain an area of active investigation (179). Connexins have now been implicated in facilitating long distance cell–cell communication *via* EVs (170, 180, 181). Cx43 has been shown to form functional hemichannels that may serve to facilitate the release of the cargo entrapped within EVs (180). Connexins in EVs may not be restricted to Cx43 as Cx46 has also been localized to EV membranes, where it may serve to enhance breast cancer cell malignancy (182). Potential clinical application of EVs may be found in drug delivery as the addition of Cx43 to doxorubicin-containing EVs reduced the cardiotoxicity of this chemotherapeutic agent (181). In another promising example, the Cx43-mimetic peptide αCT1 has shown promise in the treatment of diabetic foot and venous leg ulcers (183, 184), while a shorter version of αCT1 which lacks the antennapedia internalization sequence, known as αCT11, has been reported to exhibit cardioprotective effects (185). However, the clinical application of this drug is hindered by poor pharmacokinetic properties. As such, the encapsulation of αCT11 and other Cx43 drug candidates in EVs is being explored as a means of improving therapeutic delivery (186). In a recent example where EVs may have diagnostic potential as a biomarker, EVs released from osteoarthritis-derived chondrocytes were found to be enriched in Cx43 (187). Cx43-positive small EVs were found to promote inflammation and cell senescence that contributed to progression of osteoarthritis (187). In still another study, it was found that Cx43 selectively incorporates miRNAs into EVs implying that Cx43-positive EVs play a fundamental role in modulating distant cellular signaling (188). Potential applications of EVs in diagnostics and therapeutics is an exciting area that may have implications in numerous disease scenarios that include atrial fibrillation (189), infectious disease (190), cancer (191), and osteoarthritis (187).

## Future perspectives

Over the last half century, the connexin community has witnessed an astonishing increase in the fundamental knowledge of the cellular processes that engage the services of connexins. No longer can connexins be assigned to strictly having a role in GJIC, as the evidence for multiple roles has become overwhelming. As the breadth of cellular mechanisms that engage either full-length connexins or N-terminal truncated connexin gene products has grown, an equally huge challenge has emerged as to where, when, and how connexins engage in such a diverse set of cellular roles in human physiology and pathology. It is critically important to determine which of these cellular roles can be specifically and effectively targeted to improve cellular health and mitigate disease. The fact that at least one connexin isoform is found in nearly every human cell type point to not only their fundamental importance but also highlights the need to develop connexin customized therapeutics that can be deployed in a tissue-specific manner. Novel therapeutic targets and targeting strategies will continue to push the field of connexin biology to embrace the full gamut of canonical and noncanonical roles that were once inconceivable.

## Supporting information

This article contains supporting information.

## Conflict of interest

The authors declare that they have no conflicts of interest with the contents of this article.

## Supporting information

Supporting Table S1 Expression of human connexin isoforms in adult human organs, tissues, and cells as revealed *in situ* or primary cell culture. Table reflects a systematic analysis of articles available on PubMed, assessing cells, tissues, and organs for connexin expression in human adults. Organ and tissue expression of connexins was generated using results for the entire intact organ and it’s comprising tissues or cell types, indicated by superscripts. Organs/tissues are noted in bold. Compartmentalization of the list represents the 12 body systems. SM/SMCs, Smooth Muscle Cells.
