# Supplementary material for: Diversity in connexin biology
Source: J Biol Chem. 2023 Sep 20;299(11):105263. doi: 10.1016/j.jbc.2023.105263 (PMC10598745; doi:10.1016/j.jbc.2023.105263)
Supplement: Supporting Table S1 — Expression of human connexin isoforms in adult human organs, tissues, and cells as revealed in situ or primary cell culture. Table reflects a systematic analysis of articles available on PubMed, assessing cells, tissues, and organs for connexin expression in human adults. Organ and tissue expression of connexins was generated using results for the entire intact organ and it’s comprising tissues or cell types, indicated by superscripts. Organs/tissues are noted in bold. Compartmentalization of the list represents the 12 body systems. SM/SMCs, Smooth Muscle Cells. [file mmc1.docx]

| **Organ/Tissue/**  **Cell Type** | **Connexin** | | | | | | | | | | | | | | | | | | | | |
| --- | --- | --- | --- | --- | --- | --- | --- | --- | --- | --- | --- | --- | --- | --- | --- | --- | --- | --- | --- | --- | --- |
|  | **23** | **25** | **26** | **30** | **30.2** | **30.3** | **31** | **31.1** | **31.9** | **32** | **36** | **37** | **40** | **40.1** | **43** | **45** | **46** | **47** | **50** | **59** | **62** |
| **Heart^A^** |  |  | 1 |  | 2, 3, 4 |  |  |  | 2, 3, 9 |  |  | 5,6 | 5, 7, 8, 10 | 2 | 5, 6, 7, 8, 10, 154, 211 | 5, 7, 8, 10 | 6 |  |  |  | 2 |
| Cardiomyocytes |  |  | 1 |  |  |  |  |  |  |  |  |  | 5, 7, 8 |  | 5, 7, 8 | 5, 7, 8 |  |  |  |  |  |
| Cardiac Fibroblasts |  |  |  |  |  |  |  |  |  |  |  |  |  |  | 211 |  |  |  |  |  |  |
| Cardiac Macrophages |  |  |  |  |  |  |  |  |  |  |  |  |  |  | 154 |  |  |  |  |  |  |
| SA Nodal Cells |  |  |  |  |  |  |  |  |  |  |  |  | 10 |  |  | 10 |  |  |  |  |  |
| AV Nodal Cells |  |  |  |  |  |  |  |  | 9 |  |  |  | 8 |  | 6, 10 | 8 |  |  |  |  |  |
| Bundle of His Cells |  |  |  |  |  |  |  |  |  |  |  |  | 5 |  | 6, 10 | 5 |  |  |  |  |  |
| Purkinje Fiber Cells |  |  |  |  |  |  |  |  |  |  |  |  | 8 |  | 8 |  |  |  |  |  |  |
| **Arteries^B^** |  |  |  |  |  |  |  |  |  |  |  | 11, 12, 13, 15 | 11, 12, 13, 16, 17, 18 |  | 12, 13, 14, 17, 19, 20 | 22 |  |  |  |  |  |
| **Veins^B^** |  |  |  |  |  |  |  |  |  |  |  | 11, 12, 13, 15 | 11, 12, 13, 16, 17, 18 |  | 12, 13, 14, 17, 19, 20 |  |  |  |  |  |  |
| **Capillaries^C^** |  |  |  |  |  |  |  |  |  |  |  | 11, 12, 13 | 11, 12, 13 |  | 12, 13, 14, 21 |  |  |  |  |  |  |
| Endothelial Cells |  |  |  |  |  |  |  |  |  |  |  | 11, 12, 13 | 11, 12, 13 |  | 12, 13, 14 |  |  |  |  |  |  |
| Vascular SMCs |  |  |  |  |  |  |  |  |  |  |  | 15 | 16, 17, 18 |  | 17, 19, 20 |  |  |  |  |  |  |
| Pericytes |  |  |  |  |  |  |  |  |  |  |  |  |  |  | 21 |  |  |  |  |  |  |
|  | | | | | | | | | | | | | | | | | | | | | |
| **Lungs^D^** |  | 30 | 23 | 30, 31 | 30 | 30, 31 | 30, 31 | 30, 31 | 24 | 30, 32 |  | 30 | 30 | 30 | 25, 26, 27, 28, 29 | 28 | 30 | 30 |  | 30 |  |
| **Bronchi^E^** |  | 30 | 30, 31 | 30, 31 | 30 | 30, 31 | 30, 31 | 30, 31 |  | 30, 32 |  | 30 | 30 | 30 | 30, 31 | 30 | 30 | 30 |  | 30 |  |
| Type I Pneumocytes |  |  |  |  |  |  |  |  |  |  |  |  |  |  | 25, 26 |  |  |  |  |  |  |
| Type II Pneumocytes |  |  |  |  |  |  |  |  |  |  |  |  |  |  | 26 |  |  |  |  |  |  |
| Alveolar Macrophages |  |  |  |  |  |  |  |  |  |  |  |  |  |  | 25 |  |  |  |  |  |  |
| Pulmonary Fibroblasts |  |  |  |  |  |  |  |  |  |  |  |  |  |  | 28, 29 | 28 |  |  |  |  |  |
| **Larynx** |  |  | 34 | 34 |  |  |  |  |  | 34 |  |  |  |  | 34 |  |  |  |  |  |  |
| **Trachea^E^** |  | 30 | 30, 31 | 30, 31 | 30 | 30, 31 | 30, 31 | 30, 31 |  | 30, 32 |  | 30 | 30 | 30 | 30, 31 | 30 | 30 | 30 |  | 30 |  |
| Airway Epithelial Cells |  |  | 30, 31 | 30,31 |  | 31 | 31 | 31 |  | 30, 32 |  |  |  |  | 30, 31 |  |  |  |  |  |  |
| Airway Smooth Muscle |  |  |  |  |  |  |  |  |  |  |  |  |  |  | 33 |  |  |  |  |  |  |
| **Diaphragm^F^** |  |  |  |  |  |  |  |  |  | 193, 194 |  |  |  |  | 186, 193, 194, 195 |  |  |  |  |  |  |
|  | | | | | | | | | | | | | | | | | | | | | |
| **Brain^G^** |  |  | 35, 36 | 36, 37 | 38 |  |  |  | 4 | 38, 39, 40 | 36, 41, 46 | 36 | 36 |  | 35, 37, 40, 42, 43, 47, 48, 52 | 36 |  | 38, 39 |  |  |  |
| **Hypothalamus^H^** |  |  | 35 | 36, 37 |  |  |  |  |  | 43 | 41, 46 |  |  |  | 35, 37, 43, 45 |  |  |  |  |  |  |
| **Pituitary Gland^H^** |  |  | 35 | 36, 37 |  |  |  |  |  | 43 | 41, 46 |  |  |  | 35, 37, 43, 45 |  |  |  |  |  |  |
| **Pineal Gland** |  |  |  |  |  |  |  |  |  |  |  |  |  |  |  |  |  |  |  |  |  |
| Central Neurons |  |  |  |  |  |  |  |  |  | 35, 43 | 44 |  |  |  | 35 |  |  |  |  |  |  |
| Oligodendrocytes |  |  |  |  | 38 |  |  |  |  | 38, 39 |  |  |  |  |  |  |  | 38, 39 |  |  |  |
| Astrocytes |  |  | 35 | 36, 37 |  |  |  |  |  | 43 |  |  |  |  | 35, 37, 43, 45 |  |  |  |  |  |  |
| Microglia |  |  |  |  |  |  |  |  |  |  | 41, 46 |  |  |  | 52 |  |  |  |  |  |  |
| Ependymal Cells |  |  | 35 |  |  |  |  |  |  |  |  |  |  |  | 47, 48 |  |  |  |  |  |  |
| **Nerves^I^** |  |  |  |  |  |  |  |  |  | 51 | 49 |  |  |  | 49, 50 |  |  |  |  |  |  |
| Peripheral Neurons |  |  |  |  |  |  |  |  |  |  | 49 |  |  |  |  |  |  |  |  |  |  |
| Schwann Cells |  |  |  |  |  |  |  |  |  | 51 |  |  |  |  | 49, 50 |  |  |  |  |  |  |
| Satellite Cells |  |  |  |  |  |  |  |  |  |  |  |  |  |  | 49 |  |  |  |  |  |  |
| **Spinal Cord^J^** |  |  |  |  |  |  |  |  |  | 38, 39, 40, 53 | 36, 41, 46, 54 |  |  |  | 35, 37, 40, 42, 43, 47, 48, 52 |  |  | 38, 39 |  |  |  |
|  | | | | | | | | | | | | | | | | | | | | | |
| **Eyes^K^** |  |  | 55, 56 | 56 |  | 55, 56 | 55, 56 | 55, 56 |  | 55 | 57, 58 | 65 | 56 |  | 55, 56, 59, 60 | 55, 57 | 60 |  | 55, 60 | 56, 57 | 57 |
| **Retina** |  |  |  |  |  |  |  |  |  |  | 57, 58 |  |  |  |  | 57 |  |  |  | 57 | 57 |
| **Optic Nerve** |  |  |  |  |  |  |  |  |  |  | 57 |  |  |  |  |  |  |  |  |  | 57 |
| **Lens** |  |  |  |  |  |  |  |  |  |  |  |  |  |  | 59, 60 |  | 60 |  | 60 |  |  |
| **Cornea** |  |  | 55, 56 | 56 |  | 55, 56 | 55, 56 | 55, 56 |  | 55 |  |  | 56 |  | 55, 56 | 55 |  |  | 55 | 56 |  |
| **Ears^L^** |  |  | 61, 62, 63, 64 | 61, 62, 63, 69 |  |  |  |  |  |  |  |  |  |  | 69 |  |  |  |  |  |  |
| **Organ of Corti^M^** |  |  | 61, 62, 63 | 61, 62, 63 |  |  |  |  |  |  |  |  |  |  |  |  |  |  |  |  |  |
| Cochlear Support Cells |  |  | 61, 62, 63 | 61, 62, 63 |  |  |  |  |  |  |  |  |  |  |  |  |  |  |  |  |  |
| Fibrocytes |  |  | 61, 62, 63 | 61, 62, 63 |  |  |  |  |  |  |  |  |  |  |  |  |  |  |  |  |  |
| Spiral Ganglion Cells |  |  | 61 | 61, 69 |  |  |  |  |  |  |  |  |  |  | 69 |  |  |  |  |  |  |
| **Vestibular System^N^** |  |  | 64 |  |  |  |  |  |  | 64 |  |  |  |  |  |  |  |  |  |  |  |
| Vestibular Melanocytes |  |  | 64 |  |  |  |  |  |  | 64 |  |  |  |  |  |  |  |  |  |  |  |
| **Nose^O^** |  |  | 66 | 66 |  | 67 |  |  |  |  |  |  |  |  | 66 |  |  |  |  |  |  |
| **Olfactory Epithelium** |  |  |  |  |  |  |  |  |  |  |  |  |  |  | 68 |  |  |  |  |  |  |
| **Mouth^P^** |  |  | 70, 71, 72, 73 | 71 |  |  |  |  |  | 70, 74, 100 |  |  |  |  | 70, 72, 73, 74 | 72, 73, 74 |  |  |  |  |  |
| Oral Epithelium |  |  | 70, 73 |  |  |  |  |  |  | 70 |  |  |  |  | 70, 72 | 73 |  |  |  |  |  |
| Gingival Fibroblasts |  |  |  |  |  |  |  |  |  | 74 |  |  |  |  | 74 | 74 |  |  |  |  |  |
| **Tonsils^Q^** |  | 173 | 168 |  |  |  |  |  |  |  |  |  | 101, 165, 173 |  | 101, 165, 168 |  |  |  |  |  | 173 |
| Tonsil Epithelium |  |  | 168 |  |  |  |  |  |  |  |  |  |  |  | 168 |  |  |  |  |  |  |
| **Teeth^R^** |  |  |  |  |  |  |  |  |  | 102 |  |  | 102 |  | 75, 102 | 102 |  |  |  |  |  |
| Odontoblasts |  |  |  |  |  |  |  |  |  |  |  |  |  |  | 75 |  |  |  |  |  |  |
| **Tongue^S^** |  |  | 71 | 71 |  |  |  |  |  |  |  |  |  |  | 186 |  |  |  |  |  |  |
|  | | | | | | | | | | | | | | | | | | | | | |
| **Salivary Glands^T^** |  |  | 100 |  |  |  |  |  |  | 70, 100 |  |  |  |  | 100 |  |  |  |  |  |  |
| Serous Cells |  |  |  |  |  |  |  |  |  | 70 |  |  |  |  |  |  |  |  |  |  |  |
| Mucous Cells |  |  |  |  |  |  |  |  |  | 70 |  |  |  |  |  |  |  |  |  |  |  |
| **Pharynx** |  |  |  |  |  |  |  |  |  |  |  |  |  |  |  |  |  |  |  |  |  |
| **Esophagus** |  |  | 76, 104 |  |  |  |  |  |  |  |  |  |  |  | 76 |  |  |  |  |  |  |
| **Stomach^U^** |  |  | 77 |  |  |  |  |  |  | 78, 79, 80, 81 |  |  |  |  | 79, 81, 82, 83 |  |  |  |  |  |  |
| Gastric Epithelium |  |  | 77 |  |  |  |  |  |  | 78, 79, 80 |  |  |  |  | 79, 81, 83 |  |  |  |  |  |  |
| Gastric Circular SM |  |  |  |  |  |  |  |  |  |  |  |  |  |  | 82 |  |  |  |  |  |  |
| Gastric Longitudinal SM |  |  |  |  |  |  |  |  |  |  |  |  |  |  |  |  |  |  |  |  |  |
| **Small Intestine^V^** |  |  | 84 |  |  |  |  |  |  | 84 |  | 85 |  |  | 82, 84 |  |  |  |  |  |  |
| Small Intestine Epithelium |  |  | 84 |  |  |  |  |  |  | 84 |  | 85 |  |  | 84 |  |  |  |  |  |  |
| Small Intestine Circular SM |  |  |  |  |  |  |  |  |  |  |  |  |  |  | 82, 86 |  |  |  |  |  |  |
| Small Intestine Longitudinal SM |  |  |  |  |  |  |  |  |  |  |  |  |  |  |  |  |  |  |  |  |  |
| **Large Intestine^W^** |  | 173 | 84, 87, 88, 92 |  |  |  |  |  | 89 | 84 | 89, 92 |  | 165, 173 |  | 84, 87, 88, 90, 91, 92, 93, 165 | 89 |  |  |  |  | 173 |
| Colonic Epithelium |  |  | 87, 88 |  |  |  |  |  |  | 88 |  |  |  |  | 87, 88, 90, 91 |  |  |  |  |  |  |
| Colonic Circular SM |  |  | 87, 88 |  |  |  |  |  |  |  |  |  |  |  | 87, 88, 93 |  |  |  |  |  |  |
| Colonic Longitudinal SM |  |  | 87, 88 |  |  |  |  |  |  |  |  |  |  |  | 87, 88 |  |  |  |  |  |  |
| Colonic Interstitial Cells of Cajal |  |  | 92 |  |  |  |  |  |  |  | 92 |  |  |  | 92, 93 |  |  |  |  |  |  |
| Fibroblast-like Cells |  |  | 92 |  |  |  |  |  |  |  | 92 |  |  |  |  |  |  |  |  |  |  |
| **Rectum** |  |  |  |  |  |  |  |  |  |  |  |  |  |  |  |  |  |  |  |  |  |
| **Anus^X^** |  |  | 198, 199, 200 | 198 |  | 198, 201, 202 | 198 | 198 |  | 198, 199 |  |  | 198, 199, 207 |  | 198, 199, 200, 203, 204, 200, 205, 206, 207 | 198, 207 |  |  |  |  |  |
| **Mesentery** |  |  |  |  |  |  |  |  |  |  |  |  |  |  |  |  |  |  |  |  |  |
| **Appendix** |  |  |  |  |  |  |  |  |  |  |  |  |  |  |  |  |  |  |  |  |  |
| **Gallbladder** |  |  |  |  |  |  |  |  |  |  |  |  |  |  |  |  |  |  |  |  |  |
| **Liver^Y^** |  |  | 84, 94, 95, 96 |  | 98 |  |  |  | 98, 99 | 95, 96, 97 |  |  |  | 98 | 95, 96, 97 |  |  |  |  |  |  |
| Hepatocytes |  |  | 94, 96 |  |  |  |  |  |  | 96, 97 |  |  |  |  | 96, 97 |  |  |  |  |  |  |
| Cholangiocytes |  |  | 95 |  |  |  |  |  |  | 95 |  |  |  |  | 95 |  |  |  |  |  |  |
|  | | | | | | | | | | | | | | | | | | | | | |
| **Penis^Z^** |  |  |  |  |  |  |  |  |  |  |  |  |  |  | 105, 106 |  |  |  |  |  |  |
| Corpus Cavernosum SMCs |  |  |  |  |  |  |  |  |  |  |  |  |  |  | 105, 106 |  |  |  |  |  |  |
| **Scrotum^a^** |  |  | 198, 199, 200 | 198 |  | 198, 201, 202 | 198 | 198 |  | 198, 199 |  |  | 198, 199, 207 |  | 198, 199, 200, 203, 204, 200, 205, 206, 207 | 198, 207 |  |  |  |  |  |
| **Testes^b^** |  |  | 108, 109 |  |  |  |  |  | 110 |  |  |  |  |  | 108, 111, 112, 113, 114 |  |  |  |  | 115 |  |
| Spermatogonia |  |  |  |  |  |  |  |  |  |  |  |  |  |  | 111, 112, 113 |  |  |  |  |  |  |
| Leydig Cells |  |  |  |  |  |  |  |  |  |  |  |  |  |  | 108, 111, 113, 114 |  |  |  |  |  |  |
| Sertoli Cells |  |  | 108, 109 |  |  |  |  |  |  |  |  |  |  |  | 108, 111, 113, 114 |  |  |  |  |  |  |
| **Epididymis^c^** |  |  | 107 |  |  |  |  |  |  | 107 |  |  |  |  | 107, 118 |  |  |  |  |  |  |
| Basal Cells |  |  |  |  |  |  |  |  |  |  |  |  |  |  | 107 |  |  |  |  |  |  |
| Principal Cells |  |  |  |  |  |  |  |  |  |  |  |  |  |  | 107 |  |  |  |  |  |  |
| **Vas Deferens** |  |  |  |  |  |  |  |  |  |  |  |  |  |  |  |  |  |  |  |  |  |
| **Bulbourethral Gland** |  |  |  |  |  |  |  |  |  |  |  |  |  |  |  |  |  |  |  |  |  |
| **Seminal Vesicles** |  |  |  |  |  |  |  |  |  |  |  |  |  |  |  |  |  |  |  |  |  |
| **Prostate^d^** |  |  |  |  |  |  |  |  |  | 116, 119 |  |  | 119 |  | 117 |  |  |  |  |  |  |
| Prostate Epithelial Cells |  |  |  |  |  |  |  |  |  | 116 |  |  |  |  |  |  |  |  |  |  |  |
| Prostate Interstitial Cells |  |  |  |  |  |  |  |  |  |  |  |  |  |  | 117 |  |  |  |  |  |  |
|  | | | | | | | | | | | | | | | | | | | | | |
| **Placenta** |  |  |  |  |  |  |  |  |  | 133, 135 |  | 135 | 134, 135, 136, 137 |  | 133, 135, 136, 137 | 135 |  |  |  |  |  |
| **Uterus^e^** |  |  |  |  |  |  |  |  |  | 120, 121 |  |  | 122 |  | 120, 121, 122, 123, 124 | 122 |  |  |  |  |  |
| **Endometrium^f^** |  |  | 120, 121 |  |  |  |  |  |  | 120, 121 |  |  |  |  | 120, 121 |  |  |  |  |  |  |
| Endometrial Stromal Cells |  |  |  |  |  |  |  |  |  |  |  |  |  |  | 120, 121 |  |  |  |  |  |  |
| Glandular Epithelial Cells |  |  | 120, 121 |  |  |  |  |  |  | 120, 121 |  |  |  |  |  |  |  |  |  |  |  |
| **Myometrium^g^** |  |  |  |  |  |  |  |  |  |  |  |  | 122 |  | 122, 123, 124 | 122 |  |  |  |  |  |
| Myometrial SMCs |  |  |  |  |  |  |  |  |  |  |  |  | 122 |  | 122, 123, 124 | 122 |  |  |  |  |  |
| Myometrial Interstitial Cells |  |  |  |  |  |  |  |  |  |  |  |  |  |  | 123 |  |  |  |  |  |  |
| **Oviduct** |  |  | 125 |  |  |  |  |  |  |  |  |  |  |  | 125 |  |  |  |  |  |  |
| **Ovaries^h^** |  | 126 | 126 | 126 |  | 126 | 126 | 126 |  | 126 | 126 | 126, 127, 128, 129 | 126 |  | 126 | 126, 127 |  |  | 126 | 126 | 126 |
| Oocyte |  |  |  |  |  |  |  |  |  |  |  | 129 |  |  |  |  |  |  |  |  |  |
| Granulosa Cells |  | 126 | 126 | 126 |  | 126 | 126 | 126 |  | 126 | 126 | 126, 127, 128 | 126 |  | 126 | 126, 127 |  |  | 126 | 126 | 126 |
| **Vagina** |  |  | 138 |  |  |  |  |  |  |  |  |  |  |  | 138 |  |  |  |  |  |  |
| **Vulva** |  |  |  |  |  |  |  |  |  |  |  |  |  |  |  |  |  |  |  |  |  |
| **Cervix** |  |  | 140, 141 | 140, 141 |  |  |  |  |  | 140 |  |  |  |  | 139, 140, 141 |  |  |  |  |  |  |
| **Clitoris** |  |  |  |  |  |  |  |  |  |  |  |  |  |  |  |  |  |  |  |  |  |
| **Mammary Glands^i^** |  |  | 130, 131 | 130 |  |  |  |  |  | 130, 131 |  |  |  |  | 130, 131, 132 |  | 130 |  |  |  |  |
| Myoepithelial Cells |  |  |  | 130 |  |  |  |  |  |  |  |  |  |  | 130, 131 |  | 130 |  |  |  |  |
| Luminal Epithelial Cells |  |  | 130, 131 | 130 |  |  |  |  |  | 130, 131 |  |  |  |  | 131 |  | 130 |  |  |  |  |
| Mammary Stromal Cells |  |  |  |  |  |  |  |  |  |  |  |  |  |  | 130, 132 |  |  |  |  |  |  |
|  | | | | | | | | | | | | | | | | | | | | | |
| **Pancreas^j^** |  |  | 142, 145 |  | 143 |  | 142 | 142 | 142, 143 | 142, 143, 144 | 142 | 142 |  | 143 | 142, 143 | 142 |  |  |  |  |  |
| **Exocrine Pancreas^k^** |  |  | 142 |  |  |  |  |  | 142 | 142 |  | 142 |  |  | 142 | 142 |  |  |  |  |  |
| **Endocrine Pancreas** |  |  | 142, 145 |  |  |  | 142 | 142 | 142 | 142 | 142 | 142 |  |  | 142 | 142 |  |  |  |  |  |
| Beta Cells |  |  |  |  |  |  |  |  |  |  | 142 |  |  |  |  |  |  |  |  |  |  |
| **Thyroid** |  |  |  |  |  |  |  |  |  |  |  |  |  |  | 149 |  |  |  |  |  |  |
| **Parathyroid Glands** |  |  |  |  |  |  |  |  |  |  |  |  |  |  |  |  |  |  |  |  |  |
| **Adrenal Glands^l^** |  |  | 146 |  |  |  |  |  |  | 146 |  |  |  |  | 146, 147, 148 |  |  |  | 146 |  |  |
| **Adrenal Medulla^m^** |  |  |  |  |  |  |  |  |  |  |  |  |  |  |  |  |  |  | 146 |  |  |
| Chromaffin Cells |  |  |  |  |  |  |  |  |  |  |  |  |  |  |  |  |  |  | 146 |  |  |
| **Adrenal Cortex^n^** |  |  | 146 |  |  |  |  |  |  | 146 |  |  |  |  | 146, 147, 148 |  |  |  | 146 |  |  |
| Zona Reticularis Cells |  |  |  |  |  |  |  |  |  |  |  |  |  |  | 147, 148 |  |  |  |  |  |  |
| Zona Fasciculata Cells |  |  |  |  |  |  |  |  |  |  |  |  |  |  | 147, 148 |  |  |  |  |  |  |
| Zona Glomerulosa Cells |  |  |  |  |  |  |  |  |  |  |  |  |  |  | 148 |  |  |  |  |  |  |
|  | | | | | | | | | | | | | | | | | | | | | |
| **Kidneys^o^** |  |  | 150, 158 |  | 150 | 150 |  |  |  | 150, 151 |  | 22, 152, 153 | 22, 152, 153 | 150 | 22, 152, 153, 155, 156, 157 | 22, 152, 153 |  |  |  |  |  |
| Granular Cells |  |  |  |  |  |  |  |  |  |  |  | 22, 152, 153 | 22, 152, 153 |  | 22, 152, 153 | 22, 152, 153 |  |  |  |  |  |
| Mesangial Cells |  |  |  |  |  |  |  |  |  |  |  | 22, 152, 153 | 22, 152, 153 |  | 22, 152, 153, 155 | 22, 152, 153 |  |  |  |  |  |
| Glomerular Endothelium |  |  |  |  |  |  |  |  |  |  |  | 153 | 153 |  | 153, 155 | 153 |  |  |  |  |  |
| Parietal Layer Cells |  |  |  |  |  |  |  |  |  |  |  |  |  |  | 153, 156 |  |  |  |  |  |  |
| Podocytes |  |  |  |  |  |  |  |  |  |  |  |  |  |  | 153, 155 |  |  |  |  |  |  |
| Proximal Tubule Cells |  |  | 158 |  |  |  |  |  |  | 151 |  | 153 | 153 |  | 153, 155, 157 | 153 |  |  |  |  |  |
| Distal Tubule Cells |  |  |  |  |  |  |  |  |  |  |  | 153 | 153 |  | 153 | 153 |  |  |  |  |  |
| **Ureters** |  |  | 103 |  |  |  |  |  |  |  |  |  |  |  | 103 |  |  |  |  |  |  |
| **Bladder^p^** |  |  | 163 |  |  |  |  |  |  |  |  |  |  |  | 151, 159, 160, 161 | 151, 159, 160, 161 |  |  |  |  |  |
| Suburothelial Interstitial Cells |  |  |  |  |  |  |  |  |  |  |  |  |  |  | 151 |  |  |  |  |  |  |
| Suburothelial Myofibroblasts |  |  |  |  |  |  |  |  |  |  |  |  |  |  | 159, 162 | 159 |  |  |  |  |  |
| Detrusor SMCs |  |  |  |  |  |  |  |  |  |  |  |  |  |  | 159, 160, 161 | 159, 161 |  |  |  |  |  |
| **Urethra** |  |  |  |  |  |  |  |  |  |  |  |  |  |  | 164 |  |  |  |  |  |  |
|  | | | | | | | | | | | | | | | | | | | | | |
| **Spleen^q^** |  | 173 |  |  |  |  |  |  |  |  |  | 167 | 165, 173 |  | 165, 167, 168 |  |  | 170, 171 |  |  | 173 |
| **Lymph Nodes^r^** |  | 173 |  |  |  |  |  |  |  |  |  | 167, 169 | 165, 173 |  | 165, 168, 169, 174, 175 |  |  |  |  |  | 173 |
| **Lymphatic Vessels^s^** |  |  | 165 |  |  |  |  |  | 165 |  |  | 166, 167, 169 | 165, 166, 173 |  | 166, 165, 167, 168, 159, 172 | 166 |  | 166, 170, 171 |  |  |  |
| Lymphatic Endothelial Cells |  |  |  |  |  |  |  |  |  |  |  | 167 |  |  | 167, 168 |  |  | 170, 171 |  |  |  |
| Lymphatic SMCs |  |  |  |  |  |  |  |  |  |  |  |  |  |  | 168 | 166 |  |  |  |  |  |
| High Endothelial Venule Cells |  |  |  |  |  |  |  |  |  |  |  | 169 |  |  | 168, 169 |  |  |  |  |  |  |
| **Thymus^t^** |  | 173 |  |  |  |  |  |  |  |  |  |  | 165, 173 |  | 165, 172 |  |  |  |  |  | 173 |
| Thymus Epithelial Cells |  |  |  |  |  |  |  |  |  |  |  |  |  |  | 172 |  |  |  |  |  |  |
| **Blood Cells^u^** |  | 173 |  |  |  |  |  |  |  | 173 |  | 173, 176, 180 | 165, 173, 180 |  | 165, 173, 174, 175, 176, 178, 179, 180 |  |  |  |  |  | 173, 181 |
| B-Lymphocytes |  | 173 |  |  |  |  |  |  |  |  |  |  | 165, 173 |  | 165 |  |  |  |  |  | 173 |
| T-Lymphocytes |  | 173 |  |  |  |  |  |  |  |  |  |  | 165, 173 |  | 165 |  |  |  |  |  | 173 |
| Natural Killer Cells |  | 173 |  |  |  |  |  |  |  |  |  |  | 165, 173 |  | 165 |  |  |  |  |  | 173 |
| Dendritic Cells |  |  |  |  |  |  |  |  |  |  |  |  |  |  | 174, 175 |  |  |  |  |  |  |
| Macrophages |  |  |  |  |  |  |  |  |  |  |  | 176 |  |  | 176, 177 |  |  |  |  |  |  |
| Megakaryocytes |  | 173 |  |  |  |  |  |  |  |  |  | 173 | 173 |  |  |  |  |  |  |  | 173 |
| Eosinophils |  |  |  |  |  |  |  |  |  |  |  |  |  |  | 178 |  |  |  |  |  |  |
| Neutrophils |  |  |  |  |  |  |  |  |  |  |  | 180 | 180 |  | 180 |  |  |  |  |  |  |
| Monocytes |  | 173 |  |  |  |  |  |  |  |  |  |  | 173 |  | 176, 179 |  |  |  |  |  | 173 |
| Platelets |  |  |  |  |  |  |  |  |  | 173 |  | 173 |  |  | 173 |  |  |  |  |  | 181 |
|  | | | | | | | | | | | | | | | | | | | | | |
| **Bones^v^** |  | 173 | 197 |  |  |  |  |  | 196 | 173 |  | 173, 176, 180 | 165, 173, 180, 186 |  | 165, 173, 174, 175, 176, 178, 180, 182, 183, 184, 197 | 183, 196, 197 |  |  |  |  | 173, 181 |
| Osteoclasts |  |  |  |  |  |  |  |  |  |  |  |  |  |  | 182 |  |  |  |  |  |  |
| Osteoblasts |  |  |  |  |  |  |  |  |  |  |  |  |  |  | 183 | 183 |  |  |  |  |  |
| Osteocytes |  |  |  |  |  |  |  |  |  |  |  |  |  |  | 184, 185 |  |  |  |  |  |  |
| **Bone Marrow^w^** |  | 173 | 197 |  |  |  |  |  | 196 | 173 |  | 173, 176, 180 | 165, 173, 180 |  | 165, 173, 174, 175, 176, 178, 179, 180, 183, 197 | 183, 196, 197 |  |  |  |  | 173 |
| Bone Marrow Stromal Cells |  |  |  |  |  |  |  |  |  |  |  |  |  |  | 183 | 183 |  |  |  |  |  |
| **Skeletal Muscle** |  |  |  |  |  |  |  |  |  |  |  |  |  |  | 186 |  |  |  |  |  |  |
| **Joints^x^** |  |  | 190 |  |  |  |  |  |  | 187, 188, 190 |  |  | 187 |  | 187, 188, 189, 190, 191, 192 | 187, 190, 192 | 190 |  |  |  |  |
| **Ligaments^y^** |  |  | 190 |  |  |  |  |  |  | 187, 188, 190 |  |  | 187 |  | 187, 188, 189 | 187 | 190 |  |  |  |  |
| Ligament Fibroblasts |  |  |  |  |  |  |  |  |  | 187, 188 |  |  | 187 |  | 187, 188, 189 | 187 |  |  |  |  |  |
| Chondrocytes |  |  | 190 |  |  |  |  |  |  | 190 |  |  |  |  | 190, 191, 192 | 190, 192 | 190 |  |  |  |  |
| **Tendons^z^** |  |  |  |  |  |  |  |  |  | 193, 194 |  |  |  |  | 193, 194, 195 |  |  |  |  |  |  |
| Tenocytes |  |  |  |  |  |  |  |  |  | 193, 194 |  |  |  |  | 193, 194, 195 |  |  |  |  |  |  |
|  | | | | | | | | | | | | | | | | | | | | | |
| **Skin^*^** |  |  | 198, 199, 200 | 198 |  | 198, 201, 202 | 198 | 198 |  | 198, 199 |  |  | 198, 199, 207 |  | 198, 199, 200, 203, 204, 200, 205, 206, 207 | 198, 207 |  |  |  |  |  |
| Keratinocytes |  |  | 198, 199, 200 | 198 |  | 198, 201, 202 | 198 | 198 |  | 198, 199 |  |  | 198, 199 |  | 198, 199, 200 | 198 |  |  |  |  |  |
| Melanocytes |  |  |  |  |  |  |  |  |  |  |  |  |  |  | 203, 204 |  |  |  |  |  |  |
| Dermal Fibroblasts |  |  |  |  |  |  |  |  |  |  |  |  | 207 |  | 200, 205, 206, 207 | 207 |  |  |  |  |  |
| Sebaceous Gland Cells |  |  |  |  |  |  | 198 |  |  |  |  |  |  |  | 199 |  |  |  |  |  |  |
| Eccrine Sweat Ducts |  |  | 198, 199 |  |  |  |  |  |  |  |  |  |  |  | 199 |  |  |  |  |  |  |
| **Subcutaneous Tissue** |  |  |  |  |  |  |  |  |  |  |  |  |  |  | 208 |  |  |  |  |  |  |
| **Hair Follicle** |  |  |  | 209 |  |  | 210 |  |  |  |  |  |  |  |  |  |  |  |  |  |  |
| **Nails** |  |  |  |  |  |  |  |  |  |  |  |  |  |  |  |  |  |  |  |  |  |

Superscripts indicate the organ/tissue connexin profile was developed using results from the entire intact organ/tissue itself. The tissues/cell types that comprise the organ/tissue are indicated.

A: Cardiomyocyte, Cardiac Fibroblasts, Cardiac Macrophages, SA Nodal Cells, AV Nodal Cells, Bundle of His Cells, Purkinje Fiber Cells

B: Endothelial Cells, Vascular SMCs, Pericytes

C: Endothelial Cells, Pericytes

D: Type I Pneumocyte, Type II Pneumocyte, Alveolar Macrophages, Pulmonary Fibroblasts

E: Airway Epithelial Cells, Airway Smooth Muscle Cells

F: Skeletal Muscle, Tendon

G: Central Neurons, Oligodendrocytes, Astrocytes, Microglia, Ependymal Cells

H: Astrocytes, Microglia

I: Peripheral Neurons, Schwann Cells, Satellite Cells

J: Ependymal Cells, Oligodendrocytes, Astrocytes, Central Neurons

K: Retina, Optic Nerve, Lens, Cornea

L: Organ of Corti, Vestibular System

M: Cochlear Support Cells, Fibrocytes, Spiral Ganglion Cells

N: Vestibular Melanocytes

O: Olfactory Epithelium

P: Oral Epithelium, Gingival Fibroblasts

Q: B-Cells, Tonsil Epithelium

R: Odontoblasts

S: Skeletal Muscle

T: Serous Cells, Mucous Cells

U: Gastric Epithelium, Gastric Circular SM, Gastric Longitudinal SM

V: Small Intestine Epithelium, Small Intestine Circular SM, Small Intestine Longitudinal SM

W: B-Cell, Colonic Epithelium, Colonic Circular SM, Colonic Longitudinal SM, Colonic Interstitial Cells of Cajal, Fibroblast-Like Cell

X: Skin

Y: Hepatocytes, Cholangiocytes

Z: Corpus Cavernosum SMCs

a: Skin

b: Spermatogonia, Leydig Cells, Sertoli Cells

c: Basal Cells, Principal Cells

d: Prostate Epithelial Cells, Prostate Interstitial Cells

e: Endometrium, Myometrium

f: Endometrial Stromal Cells, Glandular Epithelial Cells

g: Myometrial SMC, Myometrial Interstitial Cells

h: Ovaries, Oocyte, Granulosa Cells

i: Myoepithelial Cells, Luminal Epithelial Cells, Mammary Stromal Cells

j: Endocrine Pancreas, Exocrine Pancreas

k: Beta Cells

l: Adrenal Cortex, Adrenal Medulla

m: Chromaffin Cells

n: Zona Reticularis Cells, Zona Fasciculata Cells, Zona Glomerulosa Cells

o: Granular Cells, Mesangial Cells, Glomerular Endothelium, Parietal Layer Cells, Podocytes, Proximal Tubule Cells, Distal Tubule Cells

p: Suburothelial Interstitial Cells, Suburothelial Myofibroblasts, Detrusor SMCs

q: B-Cells, T-Cells, Lymphatic Endothelial Cells

r: B-Cells, T-Cells, Dendritic Cells, High Endothelial Venule Cells

s: Lymphatic Endothelial Cells, Lymphatic SMCs, High Endothelial Venule Cells

t: B-Cell, T-Cell, Thymus Epithelial Cells

u: B-Cells, T-Cells, Natural Killer Cells, Dendritic Cells, Macrophages, Megakaryocytes, Eosinophils, Neutrophils, Monocytes, Platelets

v: Bone Marrow, Osteocytes

w: BM Stromal Cells, B-Cells, T-Cells, Megakaryocytes, Natural Killer Cells, Eosinophils, Neutrophils, Macrophages, Osteoclasts, Osteoblasts, Monocytes, Platelets, Bone Marrow Derived Mesenchymal Stem Cells

x: Ligament, Chondrocytes

y: Ligament Fibroblast

z: Tenocyte

* : Keratinocytes, Melanocytes, Dermal Fibroblasts

Corresponding PubMed IDs:

1. 30228305
2. 12881038
3. 12154091
4. 12176752
5. 10336839
6. 8542077
7. 18519446
8. 1118062
9. 23085482
10. 16646591
11. 7680674
12. 9617570
13. 16361362
14. 1690612
15. 17085540
16. 7485584
17. 11401829
18. 17085540
19. 17651863
20. 16442184
21. 14638741
22. 20530971
23. 31485592
24. 12176752
25. 31563569
26. 23951234
27. 31485592
28. 14656943
29. 17005044
30. 26919292
31. 17428265
32. 11784124
33. 24793171
34. 12417773
35. 10873295
36. 31374562
37. 27516431
38. 18353664
39. 15192806
40. 1846600
41. 16211561
42. 9184118
43. 11484816
44. 10462698
45. 27083773
46. Chang HY, Scemes E, Srinivas M, Lee SC, and Spray DC. (2000) Cultured human microglia express the neuronal gap junction protein, connexin36. *Soc Neurosci Abstr* ***26****:1640*
47. 31610338
48. 32312952
49. 19450429
50. 8752133
51. 30042657
52. 22417726
53. 25101702
54. 10462698
55. 24669234
56. 15914609
57. 20979653
58. 28070649
59. 223151788
60. 32038277
61. 19450429
62. 26941236
63. 28513246
64. 11169908
65. 35682601
66. 26919292
67. 17623229
68. 32506304
69. 24241398
70. 10204603
71. 17695503
72. 26893879
73. 24788723
74. 29596891
75. 12489165
76. 8207042
77. 20372813
78. 7557130
79. 8775000
80. 7670161
81. 18396685
82. 15938003
83. 24040271
84. 18267319
85. 18691394
86. 8269476
87. 23437299
88. 15704645
89. 21406965
90. 19528242
91. 17570214
92. 27916369
93. 10873019
94. 26713526
95. 30943209
96. 26648344
97. 2173931
98. 12881038
99. 12176752
100. 36144660
101. 10792506
102. 12441089
103. 8979271
104. 18288393
105. 9788101
106. 8388962
107. 22611165
108. 12210085
109. 21161545
110. 12176752
111. 10502068
112. 12649345
113. 17653298
114. Mauro V, Chevallier D, Gilleron J, Carette D, Defamie N, Gasc JM, Segretain D, and Pointis G. (2008) Aberrant cytoplasmic accumulation of connexin 43 in human testicular seminoma. *Open Biomarkers J.* **1**:20–27.
115. 12881038
116. 10079514
117. 12858352
118. 22611165
119. 8561862
120. 8567789
121. 9389559
122. 9523149
123. 18796153
124. 1332476
125. 9309385
126. 18505471
127. 14667880
128. 17531234
129. 32018041
130. 25383624
131. 8635493
132. 8391000
133. 10100001
134. 10527817
135. 15193866
136. 10527817
137. 12397213
138. 10084314
139. 27166013
140. 28492539
141. 16091133
142. 19000992
143. 12881038
144. 12154091
145. 15502644
146. 17102126
147. 7706446
148. 10690907
149. 19321193
150. 12881038
151. 12081783
152. 33172216
153. 32471989
154. 28431249
155. 9127334
156. 16820374
157. 12940936
158. 24335170
159. 21674053
160. 12215856
161. 16104929
162. 19054608
163. 18288642
164. 12081783
165. 10792506
166. 30355030
167. 22306086
168. 7560895
169. 15501244
170. 17234577
171. 20537300
172. 7875205
173. 22528526
174. 9209502
175. 9716912
176. 11834520
177. 12538692
178. 25110696
179. 1850762
180. 12525569
181. 32822477
182. 18266960
183. 8387535
184. 26091748
185. 19419324
186. 12881038
187. 12441089
188. 11145016
189. 23677649
190. 23416160
191. 24225059
192. 27116676
193. Banes AJ, Tsuzaki M, and Yamamoto J. (1996) Connexin expression is upregulated by mechanical load in avian and human tendon cells *Trans Orthop Res Soc*. **21**:1-3.
194. 17848160
195. 23212463
196. 31291594
197. 32984345
198. 11676838
199. 7518858
200. 10951247
201. 26091749
202. 11017804
203. 23344009
204. 10751145
205. 1328400
206. 21305658
207. 19320893
208. 36252698
209. 23219093
210. 11676838
211. 29696784
